# Supplementary figures and images for: Glial betaPix is essential for blood vessel development in the zebrafish brain
Source: eLife. 2026 Jan 16;14:RP106665. doi: 10.7554/eLife.106665 (PMC12810955; doi:10.7554/eLife.106665)

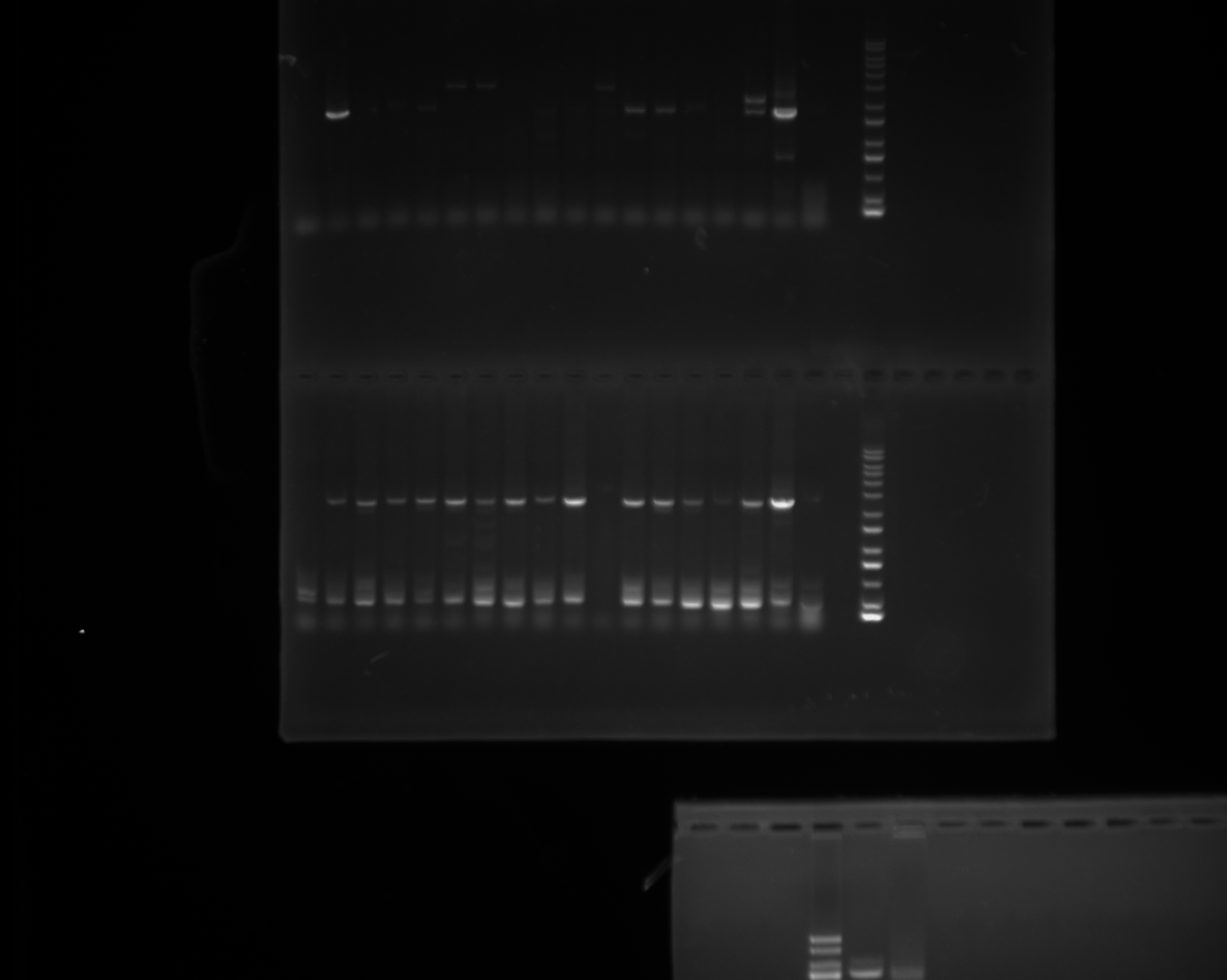

Supplement: Figure 1—source data 1. [file elife-106665-fig1-data1.zip › Figure 1-source data 1.Tif]

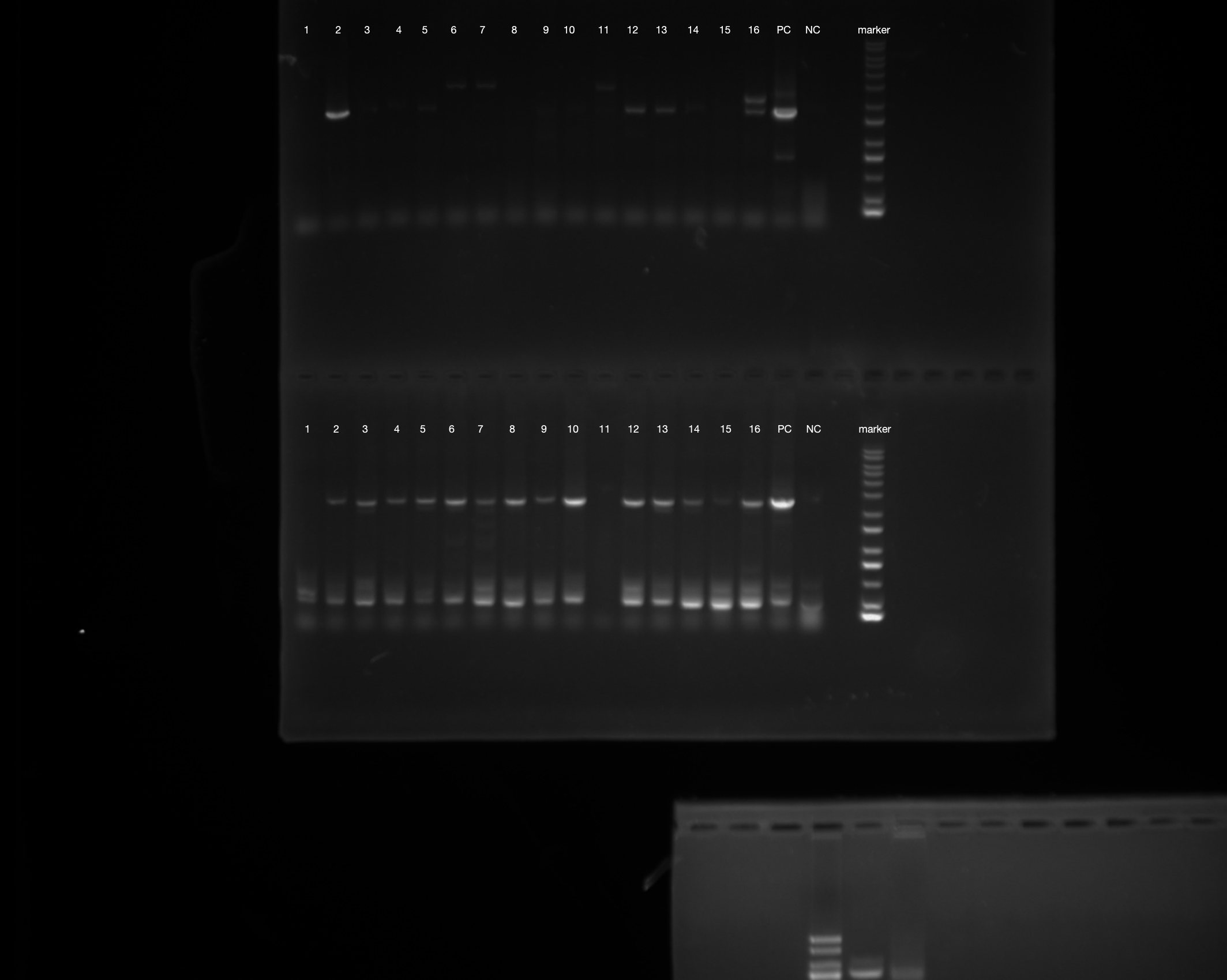

Supplement: Figure 1—source data 2. [file elife-106665-fig1-data2.zip › Figure 1-source data 2.Tif]

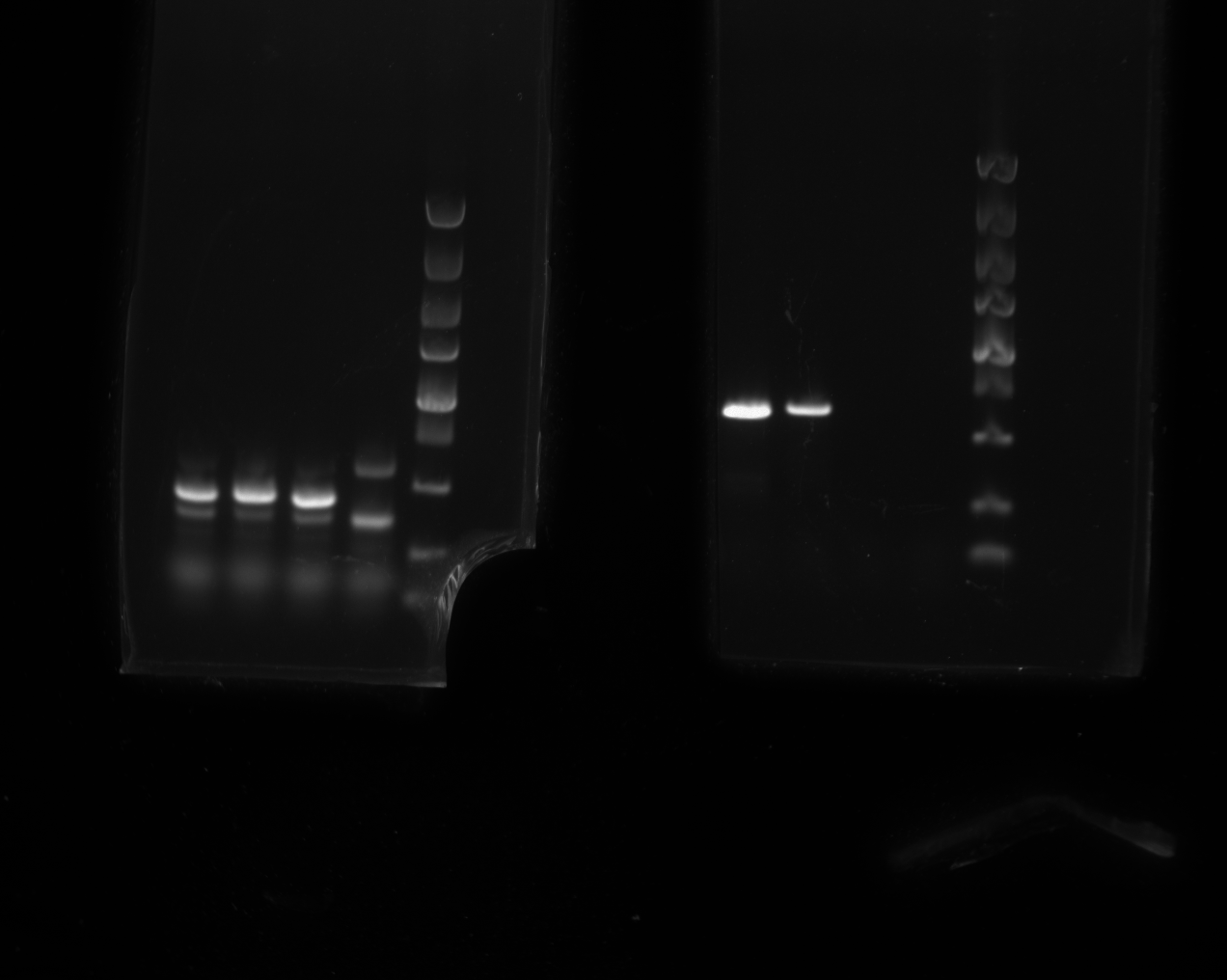

Supplement: Figure 3—figure supplement 1—source data 1. [file elife-106665-fig3-figsupp1-data1.zip › Figure 3. figure supplement 1. source data 1/For lower panel.Tif]

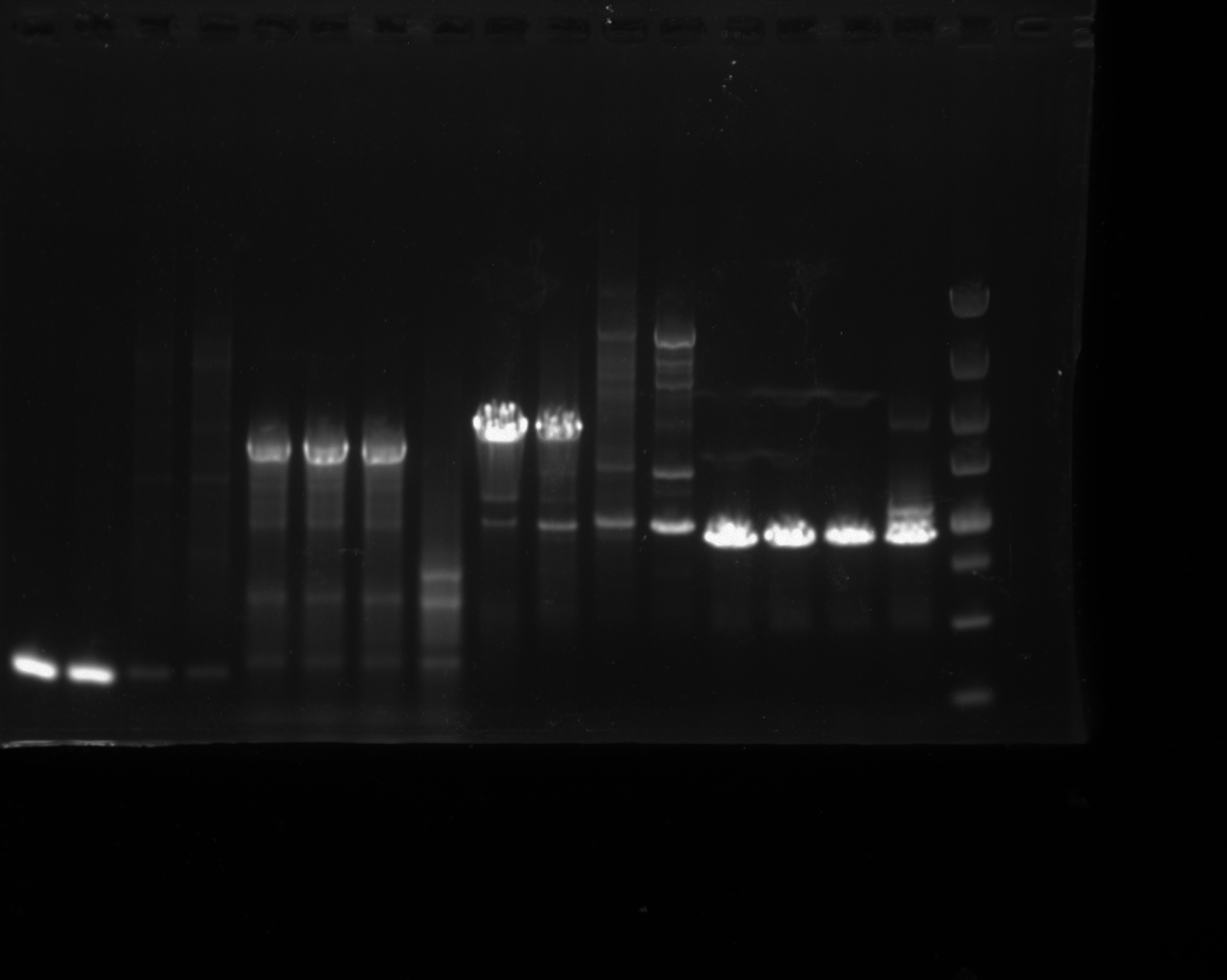

Supplement: Figure 3—figure supplement 1—source data 1. [file elife-106665-fig3-figsupp1-data1.zip › Figure 3. figure supplement 1. source data 1/For upper panel.Tif]

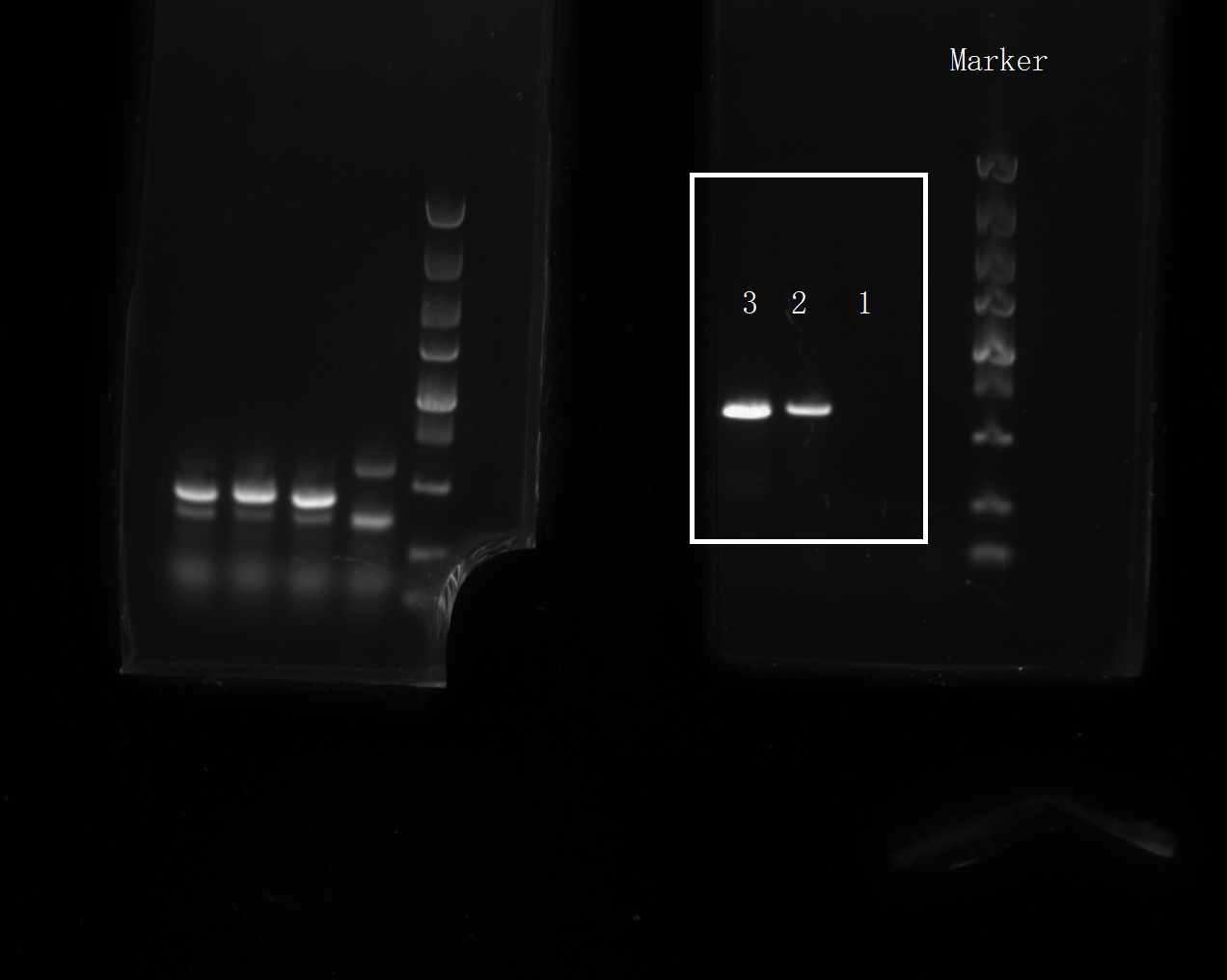

Supplement: Figure 3—figure supplement 1—source data 2. [file elife-106665-fig3-figsupp1-data2.zip › Figure 3. figure supplement 1. source data 2/For lower panel.tif]

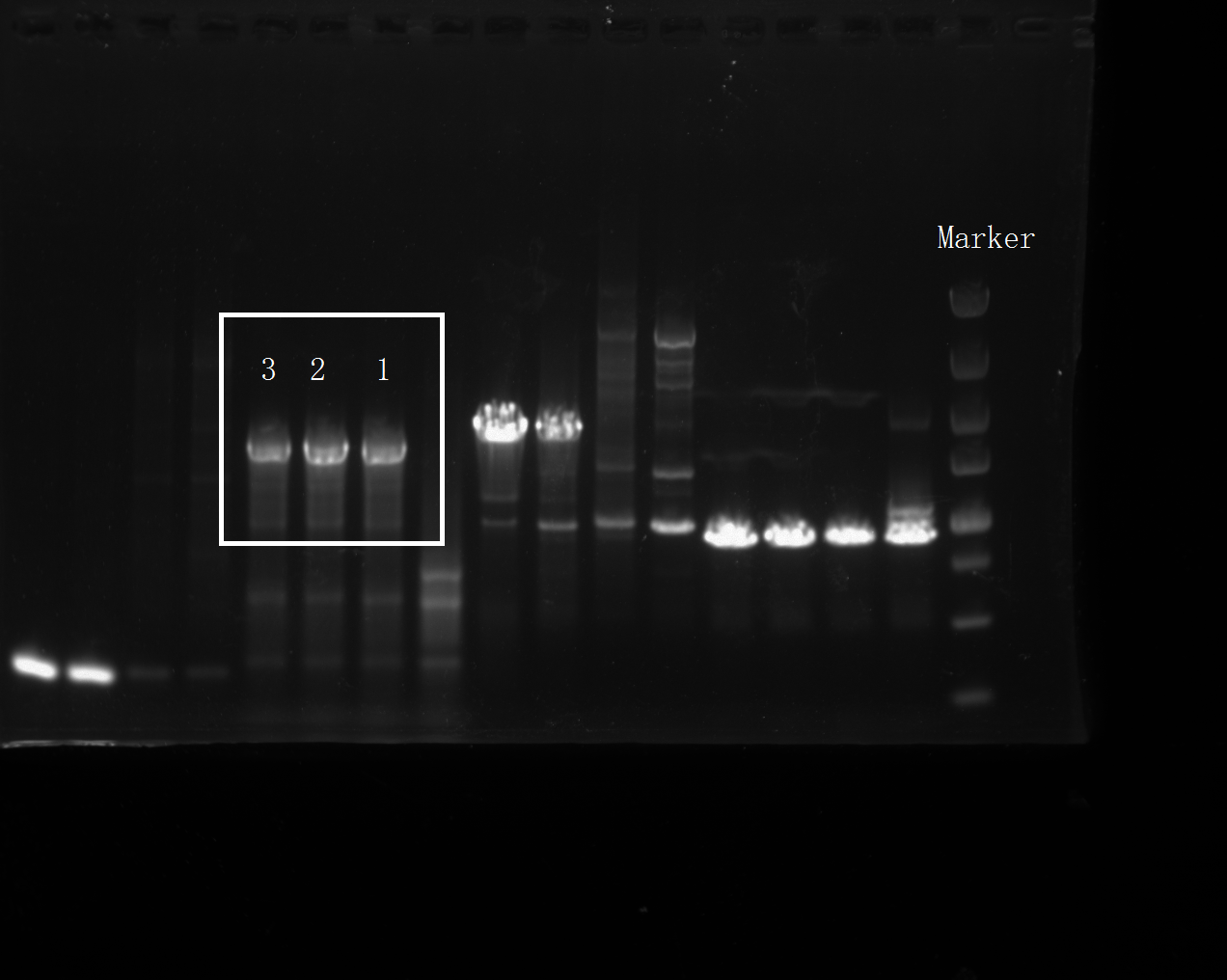

Supplement: Figure 3—figure supplement 1—source data 2. [file elife-106665-fig3-figsupp1-data2.zip › Figure 3. figure supplement 1. source data 2/For upper panel.tif]
